# Effects of fine particulate matter on bone marrow-conserved hematopoietic and mesenchymal stem cells: a systematic review

**DOI:** 10.1038/s12276-023-01149-z

**Published:** 2024-01-10

**Authors:** Govinda Bhattarai, Saroj Kumar Shrestha, Hyun-Jaung Sim, Jeong-Chae Lee, Sung-Ho Kook

**Affiliations:** 1 Department of Bioactive Material Sciences, Research Center of Bioactive Materials, Jeonbuk National University, Jeonju, 54896 Republic of Korea; 2 Cluster for Craniofacial Development and Regeneration Research, Institute of Oral Biosciences and School of Dentistry, Jeonbuk National University, Jeonju, 54896 Republic of Korea

**Keywords:** Ageing, Haematopoietic stem cells

## Abstract

The harmful effects of fine particulate matter ≤2.5 µm in size (PM_2.5_) on human health have received considerable attention. However, while the impact of PM_2.5_ on the respiratory and cardiovascular systems has been well studied, less is known about the effects on stem cells in the bone marrow (BM). With an emphasis on the invasive characteristics of PM_2.5_, this review examines the current knowledge of the health effects of PM_2.5_ exposure on BM-residing stem cells. Recent studies have shown that PM_2.5_ enters the circulation and then travels to distant organs, including the BM, to induce oxidative stress, systemic inflammation and epigenetic changes, resulting in the reduction of BM-residing stem cell survival and function. Understanding the broader health effects of air pollution thus requires an understanding of the invasive characteristics of PM_2.5_ and its direct influence on stem cells in the BM. As noted in this review, further studies are needed to elucidate the underlying processes by which PM_2.5_ disturbs the BM microenvironment and inhibits stem cell functionality. Strategies to prevent or ameliorate the negative effects of PM_2.5_ exposure on BM-residing stem cells and to maintain the regenerative capacity of those cells must also be investigated. By focusing on the complex relationship between PM_2.5_ and BM-resident stem cells, this review highlights the importance of specific measures directed at safeguarding human health in the face of rising air pollution.

## Unmasking PM2.5: the silent killer of bone marrow stem cells

Air pollution, particularly fine particulate matter (tiny particles in the air, PM2.5), poses major health dangers, including diseases linked to the breathing and heart systems, and cancer. This research by Bhattarai, Shrestha, Sim, Lee, and Kook examines how PM2.5 impacts bone marrow stem cells and hematopoietic stem cells, which are vital for blood creation and immune system operation. The scientists discovered that prolonged exposure to PM2.5 can disturb the bone marrow environment, causing oxidative stress (damage caused by free radicals) and inflammation in HSCs. This can result in irregular blood development and a weakened immune system. The research also showed that PM2.5 can adversely affect BMSCs, which are crucial for preserving the bone marrow environment and assisting tissue repair. The study emphasizes the necessity for tactics to decrease PM2.5 pollution to safeguard human health.

This summary was initially drafted using artificial intelligence, then revised and fact-checked by the author.

## Introduction

Particulate matter (PM) is a complex mixture of solid and liquid microscopic particles that enter the atmosphere as a result of natural environmental processes or human activities^1^. PM with aerodynamic diameters ≤10 µm cannot be filtered by the cilia and mucus of the nose and human respiratory tract, and instead infiltrate tracheobronchial and alveolar tissues, eventually entering the circulatory system and causing illness^2^. Fine particulate matter ≤2.5 µm in size (PM_2.5_) is especially harmful to human health^3^.

PM_2.5_ is generated from both natural environmental and human sources. The former include dust, soil particles, pollen, and sea salt aerosols, while the latter are primarily the products of combustion processes, such as fossil fuel combustion in power plants, industrial emissions, residential heating and cooking, and vehicle exhaust emissions^4^. The annual average concentration of PM_2.5_ measured in the Seoul Metropolitan area from November 2005 to March 2012 was 27 µg/m^3,^ ^5^_,_ and it was 25 µg/m^3^ in 2017^6^, which is nearly three times the WHO standard. PM_10-2.5_ is less of a concern, although these particles can irritate the eyes, nose, and throat. The substantial health risk of PM_2.5_ is related to its ability to enter the circulation and infiltrate the lungs. PM deposition in the lungs triggers airway inflammation, compromising normal immune responses and rendering this vital organ vulnerable to infection^7^. Damage to the bronchial mucociliary system induced by PM_2.5_ can impede the effective clearance of bacteria^8^. Other adverse effects of PM_2.5_ include increased inflammatory cytokine release, which triggers lung fibroblast and epithelial cell death, interference with gap junctions and thus intercellular communication, and increased permeability of the epithelial barrier, impairing its function as a physical constraint and its role in innate pulmonary immunity^9^. Following the inhalation of PM_2.5_, the particles pass through the epithelial barrier and successively cross the basement membrane, subepithelial connective tissue layer, and endothelial cells to finally enter the bloodstream^10,11^. Ambient particles are toxic to cytochrome P450s, a ubiquitous superfamily of enzymes responsible for eliminating most hydrophobic compounds in tissues throughout the body^12^. PM_2.5_ and other foreign substances can also be transferred from the circulation through pores and fenestrate in the vascular endothelium to interact with the mononuclear phagocyte system in reticular connective tissues and thereby reach potentially sensitive target sites such as the bone marrow (BM)^13,14^, where they alter the activity of stem cells. Through its direct transfer via the blood circulation to major organs, PM_2.5_ has been implicated in cardiopulmonary^15^, respiratory^16^, and cardiovascular diseases^17^, lung cancer^18^, and an increased risk of death^19,20^. PM_2.5_ is also a risk factor for asthma^21^ and, in children, for cough variant asthma by reducing immune regulation and ventilatory function^22^. Exposure to PM_2.5_ and some of its constituents is associated with a reduced hemoglobin level during the third trimester in multiparous pregnancy^23^. The BM is the major source of adult stem cells, mainly hematopoietic stem cells (HSCs) and mesenchymal stem cells (MSCs). This review particularly focuses on the impact of exposure to PM_2.5_ on BM-residing stem cells, especially HSCs and MSCs, and the underlying mechanisms.

### HSCs

HSCs are multipotent, self-renewing cells with the ability to differentiate into functional blood cells^24^. Disruption of HSC function can affect hematopoiesis, immune function, and overall health^25^. As multipotent primitive cells, HSCs can develop into all types of blood cells, including myeloid- and lymphoid-lineage cells^26^. Their maintenance is facilitated by a highly specialized niche microenvironment hosting mesenchymal stromal cells, endothelial cells, and megakaryocytes^27^. Blood cells have a high turnover rate and thus form a highly regenerative tissue. HSCs can be divided into long-term (LT)-HSCs, short-term (ST)-HSCs, and multipotent progenitor (MPP) cells. Under normal physiological conditions, LT-HSCs are in a resting state, but in response to stress, they are activated to develop into all lineage blood cells^28,29^, ensuring the long-term survival and lifelong hematopoietic function of HSCs.

HSCs undergo self-renewal, replication, and multilineage differentiation. Self-renewal is maintained by asymmetric mitosis^30^, in which one of the two daughter cells is an early progenitor cell and the other retains all of the stem cell characteristics. As a result, the number of HSCs remains constant regardless of the number of replication cycles, generating progenitor cells to meet an organism’s normal differentiation needs^31,32^. HSCs differentiate into two branches: lymphoid cells and myeloid progenitor cells. Lymphoid progenitor cells further differentiate into T cells, B cells, natural killer (NK) cells, and myeloid progenitor cells can further differentiate into red blood cells, platelets, granulocytes, macrophages, and dendritic cells^33,34^. Given the short lifespan of blood cells, HSCs must continuously differentiate to maintain their populations.

### MSCs

MSCs have self-renewal potential and tri-lineage plasticity^35^. The regulation of HSCs is mediated by MSCs, which modulate the BM microenvironment in addition to providing an osteoblastic niche. MSCs express a particular set of markers on their surface, including cluster of differentiation (CD)73, CD90, and CD105, but not CD14, CD34, CD45, and human leukocyte antigen-DR (HLA-DR)^35^. The main sources of MSCs are the BM^36^, adipose tissue^37,38^, and umbilical cord tissue^39^. BM-MSCs undergo self-renewal and multilineage differentiation^40^ into osteocytes, chondrocytes, adipocytes^37,41–44^, hepatocytes^45^, cardiomyocytes^46^, pancreatic cells^47–49^, and neuronal cells^50,51^. This differentiation potential of BM-MSCs contributes to modulation of the BM microenvironment, which can affect HSCs in a noncell-autonomous manner. Maternal exposure to PM_2.5_ during pregnancy disrupts the BM microenvironment and causes MSC senescence, followed by the senescence of HSCs and the development of myeloproliferative disease^52^.

### Mechanism of PM_2.5_ toxicity

The composition and characteristics of PM_2.5_ vary geographically and temporally depending on the source and atmospheric conditions. PM_2.5_ is mainly composed of black carbon^53^, polycyclic aromatic hydrocarbons^54,55^, aryl hydrocarbons^56^, volatile organic hydrocarbons^57^, heavy metals^58^, organic compounds^59^, minerals^60^, inorganic ions^61^, and biological materials^62^. Elements such as Al, As, Br, Ca, Cl, Cr, Fr, K, Mg, Mn, Na, Pb, Ti, and Zn, as well as sulfate, nitrate, and ammonium ions, are also commonly present in PM_2.5_^63^.

There is a relationship between exposure to airborne pollutants and poor human health^52^. In humans, the initial phase of angiohematopoiesis occurs outside of the embryo, in the yolk sac, from approximately 16 days of development^64^. Hematopoiesis is next transiently relayed in the liver before it shifts to the BM. During maternal exposure to PM_2.5_ in pregnancy, the particles directly enter the alveoli of the lungs and penetrate the blood–gas and placental barriers^65^, potentially causing premature birth and increasing the risk of mortality of preterm infants^66^.

PM_2.5_ can interfere with normal hematopoiesis and has been implicated in leukemia and other hematopoiesis-associated diseases^67^. In mice receiving an intranasal instillation of PM_2.5_ over a period of months, the number of stem cells in the BM decreased^68^. Exposure to PM_2.5_ for 2 weeks was sufficient to impair hematopoiesis in the BM of mice^69^. A summary of investigational studies on the effects of PM_2.5_ is provided in Table 1.

**Table 1:** Key findings of studies on the effects of PM_2.5_.

Studies	Key findings	References
Mice (in vivo)	PM_2.5_ exposure induces reactive oxygen species (ROS) production and oxidative stress in BM-derived endothelial progenitor cells (EPCs) and BM.	^79,160^
Mice (in vivo, in vitro)	Ni nanoparticle exposure diminishes the number and functions of EPCs in BM.	^161^
Mice (in vivo, ex-vivo)	Exposure to PM_2.5_ alters the mobilization of endothelial progenitor cells from the BM.	^162^
Adult humans, mice (in vivo)	Exposure to PM_2.5_ dramatically decreases the number of BM-derived EPCs and their activity.	^163^
Mice (in vivo, in vitro)	PM_2.5_ exposure inhibits the proliferation of BMSCs and induces apoptosis by inhibiting Akt phosphorylation, increasing ROS production, and elevating serum tumor necrosis factor (TNF)-α and interleukin (IL)-1β levels.	^68^
Nrf2^−/−^ mice (in vivo)	In wild-type mice exposed to PM_2.5_, the proportions of HSPCs, granulocyte monocyte progenitor cells (GMPs), megakaryocyte-erythrocyte progenitor cells (MEPs), and megakaryocyte progenitors (MkPs) are significantly increased. Conversely, only the proportion of HSPCs is decreased in the BM of Nrf2^−/−^ mice.	^77^
Nrf2^−/−^ mice (in vivo)	Nuclear factor erythroid-2-related Factor 2 (NRF2) controls HSPC proliferation, differentiation, and survival, plays a crucial role in myeloid formation from HSCs, and regulates HSPC retention and migration in the BM niche.	^87^
Mice (in vivo, ex vivo)	Neonates exposed to PM_2.5_ induces senescence of HSCs.	^76^
Mice (in vivo, ex vivo)	Superoxide dismutase (SOD) activity, IL-1β, IL-6, TNF-α, nuclear factor kappa light chain enhancer of activated B cells (NF-κB), and p65 increase DNA injury, and connexin 43 (Cx43) decreases DNA injury in the BM of PM_2.5_-exposed mice.	^79^
Mice (in vivo, ex vivo)	NRF2 antioxidant response genes undergo epigenetic reprogramming in the sinonasal mucosa in response to exposure to PM_2.5_.	^86^
Human placental/umbilical cord blood samples (in vitro)	Survival, proliferation, and differentiation of HSPCs are controlled by intracellular ROS levels, which are essential for preserving HSPC homeostasis.	^164^
Nrf2^−/−^ mice (in vivo)	Without controlling the quantity of ROS in the hypoxic milieu of the BM, NRF2 enhances the survival rate of HSPCs in the BM of Nrf2^−/−^ mice by preventing their apoptosis.	^165^
Mice (in vivo, in vitro)	ROS controls the self-renewal, migration, and development of HSCs and the BM microenvironment.	^166,167^
Mice (in vivo, in vitro)	NADPH oxidase 4 (NOX4) controls HSC function and is downregulated upon HSC differentiation.	^89^
Mice (in vivo, in vitro)	Pregnancy-related maternal exposure to fine PM_2.5_ causes HSC senescence with preferential disruption of the BM microenvironment, which facilitates the onset of myeloproliferative illness.	^52^
Duck (in vivo, in vitro)	Cadmium treatment significantly decreases the differentiation of BMSCs and BMMs into osteoblasts and osteoclasts and accelerates apoptosis in vitro via P2X7/PI3K/AKT signaling and the RANKL/OPG pathway, respectively.	^93^
Blood isolated from human (ex vivo)	Prolonged exposure to biomass smoke increases the risks of osteoporosis and bone resorption in premenopausal women.	^94^
Human; cohort study	Higher air pollution levels are associated with reduced bone mineral density (BMD) and a greater likelihood of osteoporosis.	^151^
BM isolated from human (in vitro)	PM_2.5_ decreases the number of BM-MSCs and increases cell death via stress-related cell shrinkage, membrane disruption, and upregulation of inflammatory markers such as TNF-α and IL-6.	^95^
Human BMSCs, human bronchial epithelial cells, and SD rats	Conditioned medium from PM_2.5_-treated 16HBE (human bronchial epithelial) cells promotes BMSC differentiation into cancer-associated fibroblasts and endothelial-like cells.	^101^
Rats (in vivo)	Repeated exposure to PM_2.5_ causes disseminated intravascular coagulation.	^120^
Human; cohort study	Short- and long-term PM_2.5_ exposure cause deep venous thrombosis and hypercoagulability.	^119^
Human; case-crossover study	Short-term exposure to PM_2.5_ induces venous thromboembolism.	^168^
Human; crossover study	Diesel exhaust inhalation increases the levels of blood markers of inflammation.	^169^
Middle-aged mice (time course study)	PM_2.5_ induces local and systemic inflammatory responses, resulting in decreased alveolar area in the lung, and an increased IL-6 level and decreased PON 1 activity in blood.	^170^
Mice (in vivo, in vitro)	BMD and bone volume decrease after prolonged exposure to organic dust, whereas trabecular spacing, serum IL-6 levels, and number of osteoclasts increase.	^158^
Human endometrial stem cells (in vitro), mice (in vivo)	PM_2.5_ exposure inhibits self-renewal, transdifferentiation, and migration in vitro and in vivo via the SERPINB2 gene.	^171^

### The mechanisms by which PM_2.5_ affect HSPCs and HSCs

Hematopoietic stem and progenitor cells (HSPCs), the progenitors of HSCs in the BM, have the capacity for self-renewal and multilineage differentiation^70^. The regulation of survival, proliferation, differentiation, and migration of HSPCs is essential for hematopoietic hemostasis and therefore the maintenance of mature immune cell generation^71–73^.

**Fig. 1:**
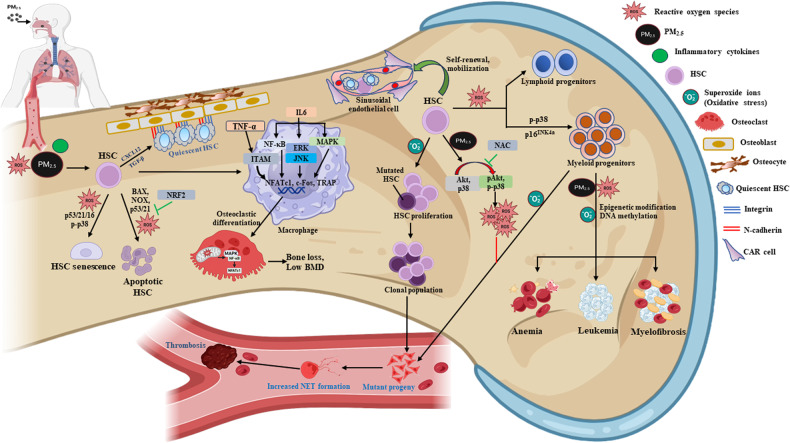
Mechanism of PM_2.5_ toxicity in BM-conserved HSCs. Inflammatory cytokines, including IL-1β, IL-6, IL-8, and TNF-α, are secreted by bronchial epithelial cells and alveolar macrophages in response to PM_2.5_ exposure. Systemic inflammatory responses are caused by the spread of these inflammatory factors from the lungs to the BM. The concentrations of PM_2.5_ and inflammatory cytokines are increased in the BM. PM_2.5_ inhibits the proliferation of HSCs, causing senescence and apoptosis. The proportion of quiescent HSCs in the BM is ~ 75%. The majority of cells are in G0 phase, and anchoring proteins on the surface of the bone, such as integrin and N-cadherin, as well as cytokines from myeloid stromal cells, such as CXCL12 and TGF-β, control the quiescence of HSCs. ROS, p53, p21, and p-p38 trigger the senescence of HSCs, whereas BAX, NOX, ROS, and p21 trigger their apoptosis. NRF2 inhibits the apoptosis of HSCs caused by PM_2.5_. In the differentiation of HSCs to macrophages, IL-6 promotes osteoclast formation in response to a low level of RANKL by controlling NF-κB, extracellular signal-regulated protein kinase/Jun N-terminal kinase, and mitogen-activated protein kinase signaling pathways, as well as transcription factors downstream of these pathways—nuclear factor-activated T-cell 1 (NFATc1), c-Fos, and tartrate resistance acid phosphatase (TRAP). TNF-α stimulates immunoreceptor tyrosine-based activation motif adaptor protein (ITAM), which promotes the transcription of NFATc1, c-Fos, and TRAP, thereby enhancing osteoclast formation and function in bone resorption. Sinusoidal endothelial (SER) cells supported by CXCL12-abundant reticular (CAR) cells in the BM regulate the proliferation, differentiation, homing, mobilization, and quiescence of HSCs. PM_2.5_ disrupts the function of sinusoid endothelial cells, resulting in a decrease in the self-renewal and proliferation abilities of HSCs and an increase in the number of quiescent HSCs. SER facilitates the differentiation of HSCs into lymphoid and myeloid progenitors. PM_2.5_ disrupts the differentiation of HSCs by decreasing the number of lymphoid progenitors and increasing the number of myeloid progenitors. Few lymphoid progenitors further differentiate into NK, T, and B cells, leading to the failure of immune functions. PM_2.5_ phosphorylates Akt and p38 in HSCs, stimulating the production of ROS and increasing oxidative stress. The increased ROS levels caused by PM_2.5_ exposure promote the excessive proliferation of myeloid progenitors, resulting in leukemia, anemia, and myelofibrosis. Superoxide ions produced by BMSCs cause mutations in HSCs, leading to the production of mutant progeny, which increases neutrophil extracellular trap (NET) formation and causes thromboses in blood vessels.

PM exposure induces the production of ROS and oxidative stress^74,75^. Both oxidative stress and inflammasome activation are enhanced in the BM of PM_2.5_-exposed mice (Fig. 1). In newborn mice, inflammasome activation can be detected in the BM for up to 12 months following PM_2.5_ inhalation, indicating that the particles enter not only the peripheral blood but also the BM^76^. Mice born after maternal PM_2.5_ exposure exhibited a similar number of lineage^-^Sca-1^+^c-Kit^+^ (LSK) cells and HSCs as control mice at 6 months of age, but at 12 months of age, the number of HSCs, but not LSK cells, was significantly increased^76^. LSK cells were highly enriched in HSPCs. In the BM, PM_2.5_ significantly increased the number of HSPCs (1.2-fold), GMPs (2.8-fold), MEPs (1.3-fold), and MkPs (1.7-fold) in wild-type mice but significantly decreased the number of HSPCs (0.8-fold) in Nrf2^−/−^ mice, suggesting that NRF2 regulates HSPC differentiation to myeloid lineages and is involved in the proliferation or survival of HSPCs^77^. The numbers of GMPs, common myeloid progenitor cells (CMPs), MEPs, and common lymphoid progenitor cells (CLPs) in the BM did not differ significantly between newborn PM_2.5_-exposed mice and control mice at 6 months^76^. However, at 12 months, the proportion of myeloid lineage cells was increased, and the proportion of lymphoid lineage cells was reduced^76^. Similarly, tertiary transplanted PM_2.5_-exposed LSK cells in the peripheral blood were shown to consist of a higher proportion of myeloid lineage cells and a lower proportion of lymphoid lineage cells^76^. A similar hematopoietic skewing toward the myeloid lineage occurs in organs repopulated by myeloid-biased (My-bi) HSCs, while skewing toward a lymphoid lineage was observed in the blood, BM, and spleen after repopulation by lymphoid-biased (Ly-bi) HSCs^78^. In another study, mice with low- and high-dose PM_2.5_ exposure exhibited pathological changes in their BM^79^. PM_2.5_ exposure was shown to decrease the proliferation of BM-derived stromal cells by inhibiting the phosphorylation of Akt (without changing apoptosis) and increasing ROS production, both of which were reversed by treatment with N-acetyl-L-cysteine (NAC) (Fig. 1)^80^. However, offspring born from NAC-treated mice showed growth retardation and had a low body weight^52^. A model simulating respiratory tract lining fluid was used to demonstrate the depletion of antioxidants in response to PM exposure^81^. In fetal mice consecutively exposed to 50 µg/m^3^ PM_2.5_ during the first 12.5 days of gestation, an increase in the levels of 8-hydroxy-2’-deoxyguanosine (8-OHdG), a major biomarker of oxidative stress, cyclooxygenase-2 (COX-2), and TNF-α, a marker of inflammation, was observed in the lungs at E16.5d. In contrast, there was no increase in mitochondrial ROS levels in the liver-derived HSCs of PM_2.5_-exposed fetal mice because PM_2.5_ inhaled during pregnancy did not directly affect these cells in the fetus^52^. BM cellularity and the mitochondrial ROS levels of BM HSCs were not significantly altered between control and PM_2.5_-exposed offspring at 2 months, but at 6 months, the BM HSCs in PM_2.5_-exposed offspring had significantly increased levels of mitochondrial ROS and senescence-associated factors, such as senescence-associated beta-galactosidase (SA-β-gal), phospho-p38, and p16^INK4a^, and increased myeloid lineage-biased differentiation, leading to functional defects in clonogenic formation and donor cell-derived repopulation, engraftment, and self-renewal potentials in transplantation experiments. The fact that the senescent phenotype of HSCs induced by maternal PM_2.5_ exposure can be reversed by NAC treatment indicates that it is triggered mainly by oxidative stress^52^. NAC is an antioxidant that reduces the levels of ROS, produces cysteine by deacetylation, and increases glutathione levels, thereby promoting the scavenging of free radicals^82^. Treatment with NAC may reduce the telomere attrition rate associated with senescence, decrease lipid peroxidation, and activate the catalytic subunit of telomerase, thereby preventing the senescence of HSCs^83^. Similar to HSCs from maternal PM_2.5_-exposed offspring, those from newborn PM_2.5_-exposed middle-aged mice showed enhanced levels of mitochondrial ROS and senescence-related functional defects, as evidenced following serial transplantation and irradiation stress^76^. The mechanism is thought to involve a deficiency of telomerase activity, leading to telomere shortening and a reduction in the reconstitution potential of HSCs during transplantation^84^. In human lung epithelial cells, PM_2.5_ causes telomerase shortening and increases the levels of senescence markers^85^. While newborn control LSK cell-transplanted recipient-derived BM cells exhibited radioprotective effects and short-term repopulation of cells and thus almost rescued all of the lethally irradiated recipient mice, newborn PM_2.5_-exposed LSK cell-transplanted recipient-derived BM cells did not exert radioprotective effects on recipient mice, and all of these mice died within 9 weeks of transplantation^76^.

Exposure to PM_2.5_ activates the NRF2 pathway^86^, a regulator of HSPCs that induces their expansion in response to stress. NRF2 modulates HSPC retention and migration in the BM microenvironment; regulates the proliferation, differentiation, and survival of HSPCs and other progenitors^87^; and plays a role in the development of myeloid cells from HSCs^88^. The level of NRF2 is increased in the BM HSCs of PM_2.5_-exposed mice and in their offspring at 6 months^52^ but is significantly decreased in NRF2-knockout mice^77^. Intracellular ROS levels regulate the survival, proliferation, and differentiation of HSPCs and are critical for maintaining their homeostasis^87,88^. NRF2 increases the survival rate of HSPCs by inhibiting their apoptosis in the BM without changing the level of ROS in the hypoxic microenvironment of the BM^88^.

NADPH oxidase (NOX)1, 2, and 4 are expressed in mouse BM LSK cells, whereas HPCs, Lin^-^ cells, and mononuclear cells from mouse BM express NOX1 and 2, but not NOX4, suggesting that the expression of NOX4 is downregulated upon HSC differentiation and that NOX4 plays an important role in regulating HSC function^89^. The expression levels of NOX4 and the NOX-associated subunits ^p67phox^, p47^phox^, and p22^phox^ are increased in the heart tissue of PM_2.5_-exposed mice^90^. Elevated ROS levels modulate HSC-specific phosphorylation of p38, limiting the function of HSCs^91^. In maternal PM_2.5_-exposed offspring, p38 expression in HSCs is upregulated. Activation of the p38 pathway contributes to the induction of p16 and cellular senescence in response to other stimuli, including DNA damage resulting from exposure to genotoxic and oxidative stress and telomere shortening due to extensive replication^92^. Compared to the BM cells from control mice, BM cells from newborn PM_2.5_-exposed 12-month-old mice exhibit reduced clonogenicity, involving colony-forming unit (CFU)- induced granulocytes/monocytes, burst-forming-unit-erythrocytes, and CFU-induced erythrocytes/macrophages/megakaryocytes. Maternal exposure to PM_2.5_ during pregnancy selectively affects the BM microenvironment, causing premature aging, with BM HSCs gradually becoming senescent via a noncell autonomous pathway. The mechanisms by which PM_2.5_ affects HSCs are shown in Fig. 1.

The above studies indicate that the effects of PM_2.5_ on mice depend on the age at exposure, with more harmful effects during the embryonic stage than the adult stage. This finding was also shown in a mouse study in which 9 of 25 (36%) 1-year-old maternal PM_2.5_-exposed offspring had a higher propensity to develop a myeloproliferative illness, with enhanced SA-gal activity in MSCs and HSCs observed over time^52^.

### The mechanisms by which PM_2.5_ affects MSCs

PM_2.5_ changes the BM microenvironment with subsequent effects on HSCs, including the induction of osteoclastogenesis and adipogenesis. The expression levels of runt-related transcription Factor 2 and osteopontin are lower in the BM cells of PM_2.5_-exposed mice than in those of control mice^76^. PM_2.5_-exposed BM cells have low bone mineralization ability and primarily differentiate into osteoclasts, both of which are closely associated with an increase in receptor activator of nuclear factor κB ligand (RANKL) levels and a decrease in osteoprotegerin (OPG) levels^76^. Exposure to cadmium significantly inhibits the differentiation of BMSCs and bone marrow macrophages (BMMs) into osteoblasts and osteoclasts and promotes apoptosis in vitro via P2X7/PI3K/AKT signaling and the RANKL/OPG system^93^. In humans, the serum level of RANKL was increased by 41% and that of OPG was reduced by 22% among 79 premenopausal women constantly exposed to PM_10-2.5_ biomass compared with control women, suggesting that chronic exposure to biomass smoke increases the risk of bone resorption and osteoporosis^94^.

**Fig. 2:**
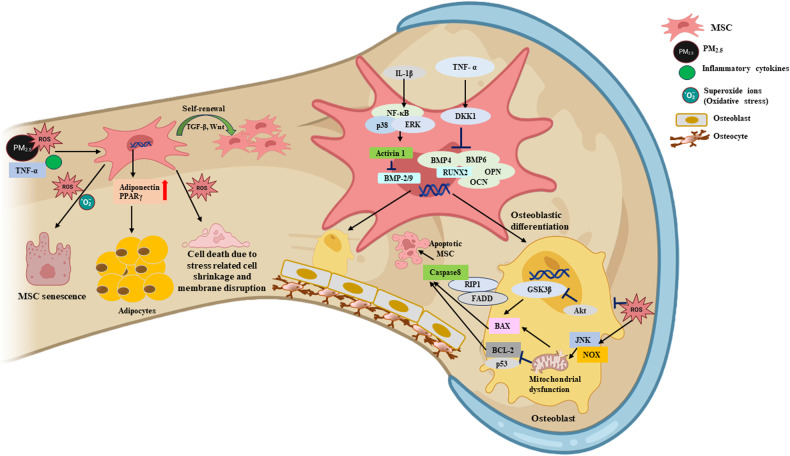
Mechanism of PM_2.5_ toxicity in BM-conserved MSCs. PM_2.5_ and inflammatory cytokines in bronchial epithelial cells enter the BM via blood vessels. PM_2.5_, inflammatory cytokines, and ROS inhibit the self-renewal and proliferation abilities of MSCs. Under normal conditions, TGF-β and Wnt facilitate the self-renewal of MSCs. ROS and oxidative stress cause the senescence of MSCs and increase their shrinkage and membrane disruption, resulting in MSC death. The production of IL-1β triggered by PM_2.5_ activates NF-κB, p38, and ERK, finally activating the Active 1 protein, which inhibits the transcription of bone morphogenic protein (BMP)-2, BMP-4, and BMP-9. These proteins are essential for the maturation of osteoblasts. TNF-α inhibits the osteogenic differentiation of MSCs by stimulating the production of Dickkopf-related protein 1 (DKK1), thereby inhibiting the transcription of osteocalcin, BMP-4, BMP-6, RUNX-2, and OPN. Furthermore, the ROS produced by MSCs after exposure to PM_2.5_ cause mitochondrial dysfunction via the Jun N-terminal kinase (JNK) and NOX pathways, leading to release of the apoptosis-promoting protein BAX and inhibition of BCL-2 production and the apoptosis of mature osteoblasts via PI3K/Akt/GSK3β signaling. TNF-α may activate FADD and bind to RIP, thereby activating caspase-8 and inducing the apoptosis of MSCs. Exposure of MSCs to PM_2.5_ stimulates the production of adiponectin and PPARγ, thereby increasing the differentiation of MSCs to adipocytes rather than osteocytes.

BM-MSCs from PM_2.5_-exposed mice show increased adipogenesis (adiponectin and peroxisome proliferator-activated receptor γ) and senescence^76^. A low concentration of PM_2.5_ increases the proliferation of BM-MSCs in vitro, but an increase in the PM_2.5_ concentration decreases the number of BM-MSCs, induces cellular morphological changes, and increases cell death caused by stress-related cell shrinkage and membrane disruption^95^. In C10 alveolar epithelial cells exposed to 50 µg PM_2.5_/cm^3^, the sub-G0/G1 phase is prolonged, indicative of both apoptotic and necrotic cell death^96^. Receptor interacting protein (RIP) and the fas-associated death domain (FADD) complex act in concert with caspase-8 and have been implicated in PM_2.5_-induced apoptosis^96^. Inflammatory markers such as TNF-α and IL-6 are upregulated in response to PM_2.5_^95^. TNF-α and FADD interact with RIP to induce apoptosis^97^. In BM-MSCs treated with 150 µg PM_2.5_/ml, antiapoptotic BCL2 is upregulated, while proapoptotic BAX and the tumor suppressor gene p53 are downregulated^95^. Exposure to PM_2.5_ promotes the secretion of inflammatory cytokines in the respiratory tract^98,99^ and promotes BM-MSC differentiation^100^ into endothelial-like cells and cancer-associated fibroblasts^101^. The levels of IL-1β, IL-6, and COX-2 mRNAs are increased in PM_2.5_-exposed 16HBE cells. IL-6 is important for inducing the expression of markers of differentiation, such as CD31, von Willebrand factor, α-smooth muscle actin (α-SMA), and fibroblast activation protein (FAP), whereas IL-1β and COX-2 induce the expression of α-SMA and FAP^101^. PM_2.5_ elevates ROS levels via NOX, a key factor in ROS generation^102^ that is also elevated in mitochondrial disorders^103^. The mechanisms by which PM_2.5_ affects MSCs are summarized in Fig. 2.

### Hematopoiesis and blood disorders related to PM_2.5_

Mesodermal progenitor cells (MPCs) differentiate into CMPs, GMPs, and other oligo-potent or unipotent progenitor cells, which in turn give rise to mature blood cells^104,105^. The effects of PM_2.5_ that have reached the BM affect BM-conserved stem cells. Children in areas with high 24 h PM_2.5_ concentrations (mean 25−50 µg/m^3^) have a higher rate of mild to moderate anemia^106^, and those in areas with very high 24 h PM_2.5_ ( >50 µg/m^3^) have a higher rate of moderate to severe anemia^95^. In animal models, PM_2.5_ exposure causes more harmful effects, including BM microenvironment impairment, in young mice than in adolescent mice^68,95^. PM_2.5_ increases ROS production and thus the cellular levels of inflammatory cytokines (TNF-α, IL-1β, and IL-6), which in turn inhibit the differentiation and proliferation of erythroid precursor cells and promote an erythropoietin-resistant state^52,68,95^. Inflammatory cytokines also cause the breakdown of ferroportin by upregulating hepcidin synthesis, thereby reducing iron absorption in the gastrointestinal tract^107,108^. Murine erythrocytes are reportedly deformed in a mice after PM_2.5_ exposure^109^. As in humans, mice exposed to air pollution have an increased risk of anemia, depending on the PM_2.5_ dose and exposure time^95^. Mice exposed to PM_2.5_ for 100 s/day for eight consecutive days have increased erythrocyte distortion, leading to hemolytic anemia (Fig. 1)^109^. Leukemia is a malignant clonal disease characterized by abnormal proliferation and impaired differentiation of HSCs. Exposure to PM_2.5_ injures HSCs in the BM^52^ and has been implicated in adult leukemia and childhood hematological malignancy^110,111^. Among the effects of long-term exposure to PM_2.5_ is the alteration of DNA methylation^112^. Molecular epidemiologic studies indicate that DNA methylation of the genes associated with leukemia positively correlates with exposure to environmental toxins^113,114^. DNA methylation involves incorporation of a methyl group at the fifth carbon of cytosine to produce 5-methylcytosine, which is oxidized to 5-hydroxymethylcytosine, a suppressor of gene expression^115,116^, which prevents cellular differentiation and thus promotes the development of leukemia. PM_2.5_ is also linked to platelet function^117,118^, and deep venous thrombosis and hypercoagulability are associated with long-term ( ~ 8 years) exposure to PM_2.5_^119^. The mechanism of PM_2.5_-associated thrombosis involves increases in the levels of inflammatory cytokines, oxidative stress, and platelet activation as well as stimulation of coagulation pathways and reduced fibrinolysis^120–122^. The increased expression levels of inflammatory cytokines, such as IL-6 and IL-1β, in rats exposed to PM_2.5_ induces disseminated intravascular coagulation^120^. The effects of moderate to high doses of PM_2.5_ include the activation of tissue-factor-dependent extrinsic pathways, coagulation, and the upregulation of adhesion molecules, such as vascular cell adhesion molecule 1 and intracellular adhesion molecule 1^118^.

### Health implications and clinical outcomes

**Fig. 3:**
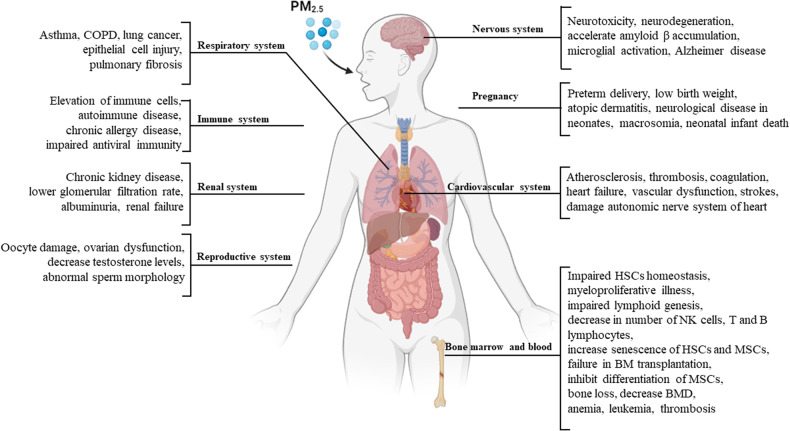
Effects of PM_2.5_ in humans. Schematic of the effects of PM_2.5_ in humans.

An increase in the incidence and mortality of certain illnesses has been linked to exposure to ambient PM_2.5_. Several studies have reported a high risk of PM_2.5_-related mortality in patients with lung cancer, ischemic heart disease, stroke, chronic obstructive pulmonary disease (COPD), and other disorders worldwide (Fig. 3)^123–128^. One of the main targets of PM_2.5_-induced toxicity is the lung, the first site of PM_2.5_ deposition in the airway. PM_2.5_ causes inflammation in the airways, impairs normal immunological responses in the lung, and increases their susceptibility to several respiratory diseases^7^. First, injury to the bronchial mucociliary system caused by PM_2.5_ suppresses the clearance of foreign particles^8^. Next, lung epithelial cells and fibroblasts undergo apoptosis due to PM_2.5_ exposure, resulting in the disruption of inflammatory cytokine networks, rendering them incapable of communicating through gap junctions. This impairment in cellular communication leads to increased permeability of the lung epithelial barrier, ultimately diminishing its efficacy as a physical component of pulmonary innate immunity^9^. PM_2.5_ reaches the lungs by inhalation and activates immune cells, such as macrophages and neutrophils, which release inflammatory mediators. Fine-tuned regulation of the survival, proliferation, differentiation, and migration of HSPCs is essential for hematopoietic homeostasis, including the normal generation of mature immune cells^71–73^. Excessive exposure to PM_2.5_ causes inflammation and the release of cytokines that act on HSPCs. Type I interferons (e.g., IFN-α) and type II interferons (e.g., IFN-γ) are important for the adaptation of HSPCs to inflammation and drive the proliferation of quiescent HSCs; however, their chronic administration impairs the self-renewal ability of HSCs^129^. This is a result of the induction of DNA damage in HSCs entering the cell cycle, which is linked to an increased mitochondrial membrane potential and mitochondrial production of ROS^130^. IL-1β is also a central mediator of innate immunity and acts directly on HSCs in vitro, promoting their proliferation and differentiation into myeloid lineage cells by activating the transcription factor PU.1. Chronic administration of IL-1β diminished the self-renewal ability of HSCs in a mouse model^131^. HSCs also respond to TNF, which induces their proliferation and directs myeloid lineage differentation in HSCs, thus compromising the repopulation of these cells^132^. The PM_2.5_-induced disruption of HSC maintenance limits the differentiation of HSCs to lymphoid-lineage progenitor cells, leading to deficiencies in NK cells and T and B lymphocytes and thus to an inability to combat infection^133^. PM_2.5_ also disrupts macrophage phagocytosis, increasing susceptibility to infection and inducing chronic lung injury^117^. Air pollution, including PM_2.5_, causes a T-cell imbalance, proinflammatory cytokine production, local pulmonary inflammation, oxidative stress, and methylation changes, all of which underlie the pathogenesis of autoimmune diseases^56^. For example, chronic exposure to PM_2.5_ causes several^134,135^ abnormalities linked to the development of type 2 diabetes mellitus (T2DM), adipose inflammation, insulin resistance (IR), and hepatic endoplasmic reticulum (ER) stress. PM_2.5_ has been suggested to modulate ER stress and inflammatory pathways, leading to IR and the development of T2DM^136^. In addition, exposure to PM_2.5_ not only causes subclinical alterations in cardiovascular function but also damages the heart’s autonomic nervous system (ANS), which decreases heart rate variability and is inextricably linked to cardiovascular morbidity and mortality^137^. Additionally, PM_2.5_ is linked to the prevalence and development of chronic kidney disease (CKD) and decreases the glomerular filtration rate (GFR)^138^. Maternal prenatal exposure to PM_2.5_ is linked to poor birth outcomes, such as preterm delivery, low birth weight, and neonatal infant death^139–141^. Furthermore, PM_2.5_ influences several other harmful health outcomes, including impaired antiviral immunity, bone loss, liver fibrosis, lung cancer, macrosomia, Alzheimer’s disease, and ovarian dysfunction (Fig. 3)^142–148^.

### Epidemiological and experimental studies

PM_2.5_ has several implications for human health, mainly related to cardiovascular and respiratory diseases and cancer^149^. Long-term exposure to PM_2.5_ was associated with a decline in bone strength among adults in Southwest China^150^ and with a decrease in BMD and an increase in the likelihood of osteoporosis^151^. PM_2.5_ at 1 µg/m^3^ increased the risk of osteoporosis by 14.6% in 8033 rural residents of Henan Province, China, between July 2015 and September 2017^152^. In several cities in Brazil from 2010 to 2018, long-term exposure to PM_2.5_ was linked to an increase in the mortality rates of a variety of cancers, including bone cancer, and persons older than 60 years had a higher risk of succumbing to bone cancer^153^. Morales-Ancajima et al. evaluated the association between hemoglobin and air pollution in a residential area among 139,368 children (ages 6–35 months). Anemia was detected in 30.8% of the exposed children, and moderate to severe anemia was detected in 8.8%^154^. Honda et al. performed a study involving 4121 older persons with anemia (34.9%) who lived in areas with high annual mean PM_2.5_ levels (>11.1 ± 2.8 μg/m^3^). Individuals with 2–5 years of exposure to PM_2.5_ pollution had a mean decline in hemoglobin levels of 0.81 ± 0.06 g/dL^155^. The PM_2.5_ levels in blood samples from adult patients (age 20–71 years) were higher in those with acute myeloid leukemia than in healthy individuals^156^. Exposure of newborn mice to PM_2.5_ induced HSC senescence by causing chronic oxidative stress and inflammasome activation in the BM^76^. Exposure of pregnant mice to PM_2.5_ caused progressive senescence of HSCs as a result of the continuous impairment of the BM microenvironment^52^. PM_2.5_ increases the numbers of systematic inflammatory cells and the risks of associated diseases in adult mice by regulating NRF2-dependent myeloid-biased hematopoiesis^77^. The proliferation of BMSCs is inhibited by PM_2.5_ exposure, leading to their degeneration and death and increasing IL-6 and TNF-α production. The osteoblastic development of BMSCs is suppressed, as is the deposition of extracellular matrix, and TNF-α, IL-6, and IL-11 mRNA levels are elevated upon coculture of chronic lymphoblastic leukemia cells with BMSCs^157^. Continuous exposure to organic dust lowered the bone density and bone volume fraction in mice and increased bone trabecular spacing and the serum IL-6 concentration and number of osteoclasts. The lung-inflammatory-bone axis, which encompasses the systemic IL-6 effector pathway, is involved in inhaled organic dust extract-mediated bone damage in mice^158^. According to Calderon et al., 6-year-old children who lived in locations with high PM_2.5_ pollution levels had decreased bone density and increased IL-6 production compared with those who resided in areas with low PM_2.5_ and air pollution levels^159^. An analysis of 175,959 men and 186,437 women showed that for each 10 µg/m^3^ increase in PM_2.5_, men and women had 17% and 14%, respectively, greater likelihoods of an increased platelet count (≥90th percentile)^117^.

## Future research

The effect of PM_2.5_ on the functional integrity of HSCs in the BM is a new avenue of investigation in research on immune disorders and blood cancers induced by PM_2.5_ exposure. However, such studies are complicated by the geographical and temporal variability of PM_2.5_ levels, which makes it difficult to determine individual exposure. Developing strategies for accurate exposure assessment, such as exposure modeling or personal monitoring approaches, would be beneficial. Among the methods through which PM_2.5_ impacts HSCs, inflammation, oxidative stress, and epigenetic alterations have been examined using cutting-edge experimental methods and molecular investigations. The identification of reliable biomarkers of PM_2.5_-induced adverse effects on HSCs is crucial for monitoring PM_2.5_ exposure in at-risk populations, analyzing PM_2.5_-derived health consequences, and evaluating therapeutic efficacy. The effects of PM_2.5_ on HSCs can be facilitated by animal models that appropriately represent human responses and by in vitro systems that accurately mimic the HSC milieu.

How long-term exposure to PM_2.5_ affects HSCs is another important area of research. Elucidating the impact of PM_2.5_ on the function, self-renewal, and differentiation potential of HSCs and thus on hematopoiesis can provide insights into the mechanism by which blood-related illnesses arise from PM_2.5_ exposure. The epigenetic changes induced by exposure to PM_2.5_ can be examined by studying DNA methylation patterns, histone modifications, and noncoding RNA expression. Epidemiological studies on the relationship between PM_2.5_ exposure and hematological diseases or anomalies in sizable populations are also essential to determine the potential hazards of PM_2.5_ and guide the development of preventative measures. All these areas of investigation should be accompanied by research on therapies and tactics that lessen the harmful effects of PM_2.5_ on HSCs, such as by determining the efficacy of protective agents, therapeutic intervention options, and air pollution reduction initiatives. Identifying susceptible groups, such as people with blood diseases or genetic predispositions, will be essential to develop focused preventative and intervention strategies.

## Conclusions

Studies on the effects of PM_2.5_ on BM-conserved MSCs and HSCs have revealed that exposure alters the BM microenvironment and causes oxidative stress, inflammation, and DNA damage in HSCs, reducing their ability to self-renew and differentiate, thereby leading to abnormal hematopoietic development. The PM_2.5_-mediated functional changes in HSCs impact the immune system and have been implicated in hematological diseases. Similar effects on BM-conserved MSCs, a cell population essential for BM microenvironment maintenance, immunological control, and tissue healing, have been linked to exposure to PM_2.5_. MSC equilibrium can be disrupted by PM_2.5_-induced oxidative stress and inflammation, resulting in decreased proliferation and poor differentiation of these cells together with weakened immunomodulatory capabilities. These results have important ramifications for MSC-mediated tissue regeneration and repair processes. The negative effects of PM_2.5_ on BM-conserved HSCs and MSCs are well established and include disturbances in hematopoiesis, the immune system, and tissue healing mechanisms. The findings summarized in this review highlight the urgent need for mitigation measures aimed at reducing PM_2.5_ pollution, the benefits of which will safeguard the structural and functional integrity of BM stem cells, including their regenerative capability. In addition to elucidating the molecular mechanisms by which PM_2.5_ exerts its detrimental effects on HSCs and MSCs, potential therapeutic interventions, such as antioxidant strategies or anti-inflammatory treatments, require increased research attention. The ability to determine the effects of PM_2.5_ on BM-conserved HSCs and MSCs in humans will encourage the development of strategies to mitigate PM_2.5_ pollution, protect human health, and advance well-being in the face of mounting environmental problems.

## References

[1] Dockery DW (1993). An association between air pollution and mortality in six US cities. N. Engl. J. Med..

[2] Lippmann M, Yeates D, Albert RJO, Medicine E (1980). Deposition, retention, and clearance of inhaled particles. Br. J. Ind. Med..

[3] Kappos AD (2004). Health effects of particles in ambient air. Int. J. Hyg. Environ. Health.

[4] Kelly FJ, Fussell JC (2015). Air pollution and public health: emerging hazards and improved understanding of risk. Environ. Geochem. Health.

[5] Lee M (2014). An analysis on the concentration characteristics of PM2.5 in Seoul, Korea from 2005 to 2012. Asia Pac. J. Atmos. Sci..

[6] Research, N.I.o.E. (National Institute of Environmental Research Incheon, Korea, (2017).

[7] Jia H (2021). PM2.5‐induced pulmonary inflammation via activating of the NLRP3/caspase‐1 signaling pathway. Environ. Toxicol..

[8] Duan Z (2013). Effects of PM2. 5 exposure on Klebsiella pneumoniae clearance in the lungs of rats. Zhonghua jie he he hu xi za zhi= Zhonghua jiehe he huxi zazhi= Chin. J. tuberculosis respiratory Dis..

[9] Longhin E (2013). Cell cycle alterations induced by urban PM2. 5 in bronchial epithelial cells: characterization of the process and possible mechanisms involved. Part. Fibre Toxicol..

[10] Jin X-T (2016). Progression and inflammation of human myeloid leukemia induced by ambient PM 2.5 exposure. Arch. Toxicol..

[11] Blank, F., von Garnier, C., Gehr, P. & Rothen-Rutishauser, B. Translocation across the air-blood tissue barrier. (CRC Press, 2014).

[12] Reed JR, dela Cruz ALN, Lomnicki SM, Backes WL (2015). Environmentally persistent free radical-containing particulate matter competitively inhibits metabolism by cytochrome P450 1A2. Toxicol. Appl. Pharmacol..

[13] Oberdörster G, Oberdörster E, Oberdörster J (2005). Nanotoxicology: an emerging discipline evolving from studies of ultrafine particles. Environ. Health Perspect..

[14] Casals E, Vázquez-Campos S, Bastús NG, Puntes V (2008). Distribution and potential toxicity of engineered inorganic nanoparticles and carbon nanostructures in biological systems. TrAC Trends Anal. Chem..

[15] Xu J-J (2013). Relationship between PM 2. 5 exposure and pulmonary function in different working environments. J. Environ. Health.

[16] Xing Y-F, Xu Y-H, Shi M-H, Lian Y-X (2016). The impact of PM2. 5 on the human respiratory system. J. Thorac. Dis..

[17] Cheng Y (2016). Ambient PM2. 5 during pregnancy and risk on preterm birth. Zhonghua liu xing bing. xue za zhi= Zhonghua liuxingbingxue zazhi.

[18] Shu Y (2016). Analysis of the relationship between PM2. 5 and lung cancer based on protein-protein interactions. Combinatorial Chem. high. throughput Screen..

[19] Wu J, Zhu J, Li W, Xu D, Liu J (2017). Estimation of the PM 2.5 health effects in China during 2000–2011. Environ. Sci. Pollut. Res..

[20] Shi T (2015). Association between PM2. 5 air pollution and daily resident mortality in Guangzhou urban area in winter. J. Environ. Health.

[21] Chen Y, Wong GW, Li J (2016). Environmental exposure and genetic predisposition as risk factors for asthma in China. Allergy, Asthma Immunol. Res..

[22] Zhang Y (2016). Correlational study on atmospheric concentrations of fine particulate matter and children cough variant asthma. Eur. Rev. Med. Pharm. Sci..

[23] Xie G (2022). Effects of PM2. 5 and its constituents on hemoglobin during the third trimester in pregnant women. Environ. Sci. Pollut. Res..

[24] Seita J, Weissman IL (2010). Hematopoietic stem cell: self‐renewal versus differentiation. Wiley Interdiscip. Rev.: Syst. Biol. Med..

[25] Verovskaya EV, Dellorusso PV, Passegué E (2019). Losing sense of self and surroundings: hematopoietic stem cell aging and leukemic transformation. Trends Mol. Med..

[26] Till JE, McCulloch EA (2012). A direct measurement of the radiation sensitivity of normal mouse bone marrow cells. Radiat. Res..

[27] Wei Q, Frenette PS (2018). Niches for hematopoietic stem cells and their progeny. Immunity.

[28] Yamada T, Park CS, Lacorazza HD (2013). Genetic control of quiescence in hematopoietic stem cells. Cell Cycle.

[29] Cabezas-Wallscheid N (2017). Vitamin A-retinoic acid signaling regulates hematopoietic stem cell dormancy. Cell.

[30] Shenghui H, Nakada D, Morrison SJ (2009). Mechanisms of stem cell self-renewal. Annu. Rev. Cell Developmental.

[31] Hinge A (2020). Asymmetrically segregated mitochondria provide cellular memory of hematopoietic stem cell replicative history and drive HSC attrition. Cell Stem Cell.

[32] Inaba M, Yamashita YM (2012). Asymmetric stem cell division: precision for robustness. Cell Stem Cell.

[33] Suda T, Arai F, Hirao A (2005). Hematopoietic stem cells and their niche. Trends Immunol..

[34] Majeti R, Park CY, Weissman IL (2007). Identification of a hierarchy of multipotent hematopoietic progenitors in human cord blood. Cell Stem Cell.

[35] Dominici M (2006). Minimal criteria for defining multipotent mesenchymal stromal cells. The International Society for Cellular Therapy position statement. Cytotherapy.

[36] Pittenger MF (1999). Multilineage potential of adult human mesenchymal stem cells. Science.

[37] Wagner W (2005). Comparative characteristics of mesenchymal stem cells from human bone marrow, adipose tissue, and umbilical cord blood. Exp. Hematol..

[38] Zhang X (2006). Runx2 overexpression enhances osteoblastic differentiation and mineralization in adipose-derived stem cells in vitro and in vivo. Calcif. tissue Int..

[39] Kita K, Gauglitz GG, Phan TT, Herndon DN, Jeschke MG (2010). Isolation and characterization of mesenchymal stem cells from the sub-amniotic human umbilical cord lining membrane. Stem Cells Dev..

[40] Ullah I, Subbarao RB, Rho GJ (2015). Human mesenchymal stem cells-current trends and future prospective. Biosci. Rep..

[41] Ranera B (2013). Expansion under hypoxic conditions enhances the chondrogenic potential of equine bone marrow-derived mesenchymal stem cells. Vet. J..

[42] Zhang X (2011). Isolation and characterization of mesenchymal stem cells from human umbilical cord blood: reevaluation of critical factors for successful isolation and high ability to proliferate and differentiate to chondrocytes as compared to mesenchymal stem cells from bone marrow and adipose tissue. J. Cell. Biochem..

[43] Gronthos S, Graves S, Ohta S, Simmons P (1994). The STRO-1+ fraction of adult human bone marrow contains the osteogenic precursors. Blood.

[44] Muruganandan S, Roman A, Sinal C (2009). Adipocyte differentiation of bone marrow-derived mesenchymal stem cells: cross talk with the osteoblastogenic program. Cell. Mol. life Sci..

[45] Stock P, Brückner S, Winkler S, Dollinger MM, Christ B (2014). Human bone marrow mesenchymal stem cell-derived hepatocytes improve the mouse liver after acute acetaminophen intoxication by preventing progress of injury. Int. J. Mol. Sci..

[46] Xu W (2004). Mesenchymal stern cells from adult human bone marrow differentiate into a cardiomyocyte phenotype in vitro. Exp. Biol. Med..

[47] Tang D-Q (2012). In vitro generation of functional insulin-producing cells from human bone marrow-derived stem cells, but long-term culture running risk of malignant transformation. Am. J. Stem Cells.

[48] Phadnis SM (2011). Human bone marrow-derived mesenchymal cells differentiate and mature into endocrine pancreatic lineage in vivo. Cytotherapy.

[49] Gabr MM (2013). Insulin-producing cells from adult human bone marrow mesenchymal stem cells control streptozotocin-induced diabetes in nude mice. Cell Transplant..

[50] Barzilay R, Ben-Zur T, Bulvik S, Melamed E, Offen D (2009). Lentiviral delivery of LMX1a enhances dopaminergic phenotype in differentiated human bone marrow mesenchymal stem cells. Stem Cells Dev..

[51] Wilkins A (2009). Human bone marrow-derived mesenchymal stem cells secrete brain-derived neurotrophic factor which promotes neuronal survival in vitro. Stem Cell Res..

[52] Bhattarai G (2020). Maternal exposure to fine particulate matter during pregnancy induces progressive senescence of hematopoietic stem cells under preferential impairment of the bone marrow microenvironment and aids development of myeloproliferative disease. Leukemia.

[53] Bové H (2019). Ambient black carbon particles reach the fetal side of human placenta. Nat. Commun..

[54] Leachi HFL (2020). Polycyclic aromatic hydrocarbons and development of respiratory and cardiovascular diseases in workers. Rev. Bras. Enferm..

[55] Alegbeleye OO, Opeolu BO, Jackson VA (2017). Polycyclic aromatic hydrocarbons: a critical review of environmental occurrence and bioremediation. Environ. Manag..

[56] Zhao C-N (2019). Emerging role of air pollution in autoimmune diseases. Autoimmun. Rev..

[57] Puri P, Nandar SK, Kathuria S, Ramesh V (2017). Effects of air pollution on the skin: A review. Indian J. Dermatol. Venereol. Leprol..

[58] Ashmore, M.R. et al. World Atlas of Atmospheric Pollution, 77–94 (2008).

[59] Grzywa-Celińska A, Krusiński A, Milanowski J (2020). ‘Smoging kills’-effects of air pollution on human respiratory system. Ann. Agric. Environ. Med..

[60] Kitamura H (2018). Impact of secondary generated minerals on toxic element immobilization for air pollution control fly ash of a municipal solid waste incinerator. Environ. Sci. Pollut. Res..

[61] Švédová B (2020). Concentration variability of water-soluble ions during the acceptable and exceeded pollution in an industrial region. Int. J. Environ. Res. Public Health.

[62] Shahrbaf MA, Akbarzadeh MA, Tabary M, Khaheshi I (2021). Air pollution and cardiac arrhythmias: A comprehensive review. Curr. Probl. Cardiol..

[63] Bangar V, Mishra AK, Jangid M, Rajput P (2021). Elemental characteristics and source-apportionment of PM2. 5 during the post-monsoon season in Delhi, India. Front. Sustain. Cities.

[64] Luckett WP (1978). Origin and differentiation of the yolk sac and extraembryonic mesoderm in presomite human and rhesus monkey embryos. Am. J. Anat..

[65] Onoda A, Takeda K, Umezawa M (2017). Dose-dependent induction of astrocyte activation and reactive astrogliosis in mouse brain following maternal exposure to carbon black nanoparticle. Part. Fibre Toxicol..

[66] Lelieveld J, Evans JS, Fnais M, Giannadaki D, Pozzer A (2015). The contribution of outdoor air pollution sources to premature mortality on a global scale. Nature.

[67] Puett RC (2020). Relationship of leukaemias with long-term ambient air pollution exposures in the adult Danish population. Br. J. Cancer.

[68] Cui Y (2015). Ambient fine particulate matter induces apoptosis of endothelial progenitor cells through reactive oxygen species formation. Cell. Physiol. Biochem..

[69] Ge J (2020). Combined exposure to formaldehyde and PM2. 5: Hematopoietic toxicity and molecular mechanism in mice. Environ. Int..

[70] Kaushansky K (2006). Lineage-specific hematopoietic growth factors. N. Engl. J. Med..

[71] Chavakis T, Mitroulis I, Hajishengallis G (2019). Hematopoietic progenitor cells as integrative hubs for adaptation to and fine-tuning of inflammation. Nat. Immunol..

[72] Ogawa M (1993). Differentiation and proliferation of hematopoietic stem cells. Blood.

[73] Wright DE, Wagers AJ, Gulati AP, Johnson FL, Weissman IL (2001). Physiological migration of hematopoietic stem and progenitor cells. Science.

[74] Khurshid SS, Siegel JA, Kinney KA (2014). Indoor particulate reactive oxygen species concentrations. Environ. Res..

[75] Daher N (2014). Oxidative potential and chemical speciation of size-resolved particulate matter (PM) at near-freeway and urban background sites in the greater Beirut area. Sci. Total Environ..

[76] Bhattarai G, Sim H-J, So H-S, Lee J-C, Kook S-H (2023). Exposure of newborns to atmospherically relevant artificial particulate matter induces hematopoietic stem cell senescence. J. Hazard. Mater..

[77] Wang Y (2023). PM2. 5 Increases Systemic Inflammatory Cells and Associated Disease Risks by Inducing NRF2-Dependent Myeloid-Biased Hematopoiesis in Adult Male Mice. Environ. Sci. Technol..

[78] Muller-Sieburg CE, Cho RH, Karlsson L, Huang J-F, Sieburg HB (2004). Myeloid-biased hematopoietic stem cells have extensive self-renewal capacity but generate diminished lymphoid progeny with impaired IL-7 responsiveness. Blood.

[79] Liu, L.-L. et al. Effects of Long-Term Exposure to PM2. 5 on Oxidative Stress Injury and Expression of Inflammatory Factors, NF-κB p65 and Cx43 in Bone Marrow of Mice. **10**, 747286 (2022).

[80] Cui Y (2015). Ambient fine particulate matter suppresses in vivo proliferation of bone marrow stem cells through reactive oxygen species formation. PLoS One.

[81] Godri KJ (2010). Particulate oxidative burden associated with firework activity. Environ. Sci. Technol..

[82] Banaclocha MM, Hernandez AI, Martınez N, Ferrandiz ML (1997). N-acetylcysteine protects against age-related increase in oxidized proteins in mouse synaptic mitochondria. Brain Res..

[83] Voghel G (2008). Chronic treatment with N-acetyl-cystein delays cellular senescence in endothelial cells isolated from a subgroup of atherosclerotic patients. Mech. Ageing Dev..

[84] Samper E (2002). Long-term repopulating ability of telomerase-deficient murine hematopoietic stem cells. Blood, J. Am. Soc. Hematol..

[85] Chang-Chien J (2021). Particulate matter causes telomere shortening and increase in cellular senescence markers in human lung epithelial cells. Ecotoxicol. Environ. Saf..

[86] Park, B. et al. In International Forum of Allergy & Rhinology, Vol. 12 1424 (Wiley-Blackwell, 2022). 10.1002/alr.23010 PMC9790744 35426488

[87] Tsai JJ (2013). Nrf2 regulates haematopoietic stem cell function. Nat. Cell Biol..

[88] Dai X (2020). Nrf2: redox and metabolic regulator of stem cell state and function. Trends Mol. Med.

[89] Wang Y (2010). Total body irradiation causes residual bone marrow injury by induction of persistent oxidative stress in murine hematopoietic stem cells. Free Radic. Biol. Med.

[90] Wu F, Zhang J (2018). The involvement of Nox4 in fine particulate matter exposure-induced cardiac injury in mice. J. Toxicological Sci..

[91] Ito K (2006). Reactive oxygen species act through p38 MAPK to limit the lifespan of hematopoietic stem cells. Nat. Med..

[92] Iwasa H, Han J, Ishikawa F (2003). Mitogen‐activated protein kinase p38 defines the common senescence‐signalling pathway. Genes Cells.

[93] Ma Y (2021). Cadmium exposure triggers osteoporosis in duck via P2X7/PI3K/AKT-mediated osteoblast and osteoclast differentiation. Sci. Total Environ..

[94] Saha H, Mukherjee B, Bindhani B, Ray MR (2016). Changes in RANKL and osteoprotegerin expression after chronic exposure to indoor air pollution as a result of cooking with biomass fuel. J. Appl Toxicol..

[95] Abu-Elmagd M (2017). Evaluation of the effects of airborne particulate matter on bone marrow-mesenchymal stem cells (BM-MSCs): cellular, molecular and systems biological approaches. Int. J. Environ. Res. Public Health.

[96] Altekruse, S. SEER cancer statistics review, 1975–2007. http://seer.cancer.gov/csr/1975_2007/results_merged/sect_13_leukemia.pdf (2009).

[97] Humphries F, Yang S, Wang B, Moynagh PN (2015). RIP kinases: key decision makers in cell death and innate immunity. Cell Death Differ..

[98] Lee M-S (2014). Oxidative stress and systemic inflammation as modifiers of cardiac autonomic responses to particulate air pollution. Int. J. Cardiol..

[99] Corsini E (2013). Comparison of wood smoke PM2. 5 obtained from the combustion of FIR and beech pellets on inflammation and DNA damage in A549 and THP-1 human cell lines. Arch. Toxicol..

[100] Ostro B (2014). Chronic PM2. 5 exposure and inflammation: determining sensitive subgroups in mid-life women. Environ. Res..

[101] Jin X, Su R, Li R, Cheng L, Li Z (2018). Crucial role of pro-inflammatory cytokines from respiratory tract upon PM2. 5 exposure in causing the BMSCs differentiation in cells and animals. Oncotarget.

[102] Lambeth JD (2004). NOX enzymes and the biology of reactive oxygen. Nat. Rev. Immunol..

[103] Jin X (2016). Amelioration of particulate matter-induced oxidative damage by vitamin C and quercetin in human bronchial epithelial cells. Chemosphere.

[104] Manz MG, Boettcher S (2014). Emergency granulopoiesis. Nat. Rev. Immunol..

[105] Jacobsen SEW, Nerlov C (2019). Haematopoiesis in the era of advanced single-cell technologies. Nat. Cell Biol..

[106] Organization, W. H. Ambient air pollution: A global assessment of exposure and burden of disease. (2016).

[107] Ferrucci, L. & Balducci, L. In Seminars in hematology, Vol. 45 242–249 (Elsevier, 2008). 10.1053/j.seminhematol.2008.06.001 PMC2645640 18809094

[108] Kido T (2011). Particulate matter induces translocation of IL-6 from the lung to the systemic circulation. Am. J. Respir. Cell Mol. Biol..

[109] Wardoyo AY, Juswono UP, Noor JA (2019). How exposure to ultrafine and fine particles of car smoke can alter erythrocyte forms of male mice. Pol. J. Environ. Stud..

[110] Lavigne É (2017). Maternal exposure to ambient air pollution and risk of early childhood cancers: a population-based study in Ontario, Canada. Environ. Int..

[111] Hvidtfeldt UA (2020). Residential exposure to PM2. 5 components and risk of childhood non-hodgkin lymphoma in Denmark: a nationwide register-based case-control Study. Int. J. Environ. Res. Public Health.

[112] Sanchez-Guerra M (2015). Effects of particulate matter exposure on blood 5-hydroxymethylation: results from the Beijing truck driver air pollution study. Epigenetics.

[113] Cortessis VK (2012). Environmental epigenetics: prospects for studying epigenetic mediation of exposure–response relationships. Hum. Genet..

[114] Hou L, Zhang X, Wang D, Baccarelli A (2012). Environmental chemical exposures and human epigenetics. Int. J. Epidemiol..

[115] Madakashira BP, Sadler KC (2017). DNA methylation, nuclear organization, and cancer. Front. Genet..

[116] Koch A (2018). Analysis of DNA methylation in cancer: location revisited. Nat. Rev. Clin. Oncol..

[117] Zhang Z (2018). Long-term exposure to ambient particulate matter (PM2. 5) is associated with platelet counts in adults. Environ. Pollut..

[118] Robertson S, Miller MR (2018). Ambient air pollution and thrombosis. Part. Fibre Toxicol..

[119] Brook RD (2010). Particulate matter air pollution and cardiovascular disease: an update to the scientific statement from the American Heart Association. Circulation.

[120] Liang S (2019). Repeat dose exposure of PM2. 5 triggers the disseminated intravascular coagulation (DIC) in SD rats. Sci. Total Environ..

[121] Rückerl R (2014). Associations between ambient air pollution and blood markers of inflammation and coagulation/fibrinolysis in susceptible populations. Environ. Int..

[122] Hajat A (2015). Long-term exposure to air pollution and markers of inflammation, coagulation, and endothelial activation: a repeat-measures analysis in the Multi-Ethnic Study of Atherosclerosis (MESA). Epidemiol. (Camb., Mass.).

[123] Hystad P (2020). Associations of outdoor fine particulate air pollution and cardiovascular disease in 157 436 individuals from 21 high-income, middle-income, and low-income countries (PURE): a prospective cohort study. Lancet Planet Health.

[124] Gao X (2021). Short-term exposure to PM2. 5 components and renal health: Findings from the Veterans Affairs Normative Aging Study. J. Hazard Mater..

[125] Liu L, Zhang Y, Yang Z, Luo S, Zhang Y (2021). Long-term exposure to fine particulate constituents and cardiovascular diseases in Chinese adults. J. Hazard Mater..

[126] Lao XQ (2019). Long-term exposure to ambient fine particulate matter (PM 2.5) and incident type 2 diabetes: a longitudinal cohort study. Diabetologia.

[127] Gilcrease GW (2020). Is air pollution affecting the disease activity in patients with systemic lupus erythematosus? State of the art and a systematic literature review. Eur. J. Rheumatol..

[128] Pun VC, Kazemiparkouhi F, Manjourides J, Suh HH (2017). Long-term PM2. 5 exposure and respiratory, cancer, and cardiovascular mortality in older US adults. Am. J. Epidemiol..

[129] Essers MA (2009). IFNα activates dormant haematopoietic stem cells in vivo. Nature.

[130] Walter D (2015). Exit from dormancy provokes DNA-damage-induced attrition in haematopoietic stem cells. Nature.

[131] Pietras EM (2016). Chronic interleukin-1 exposure drives haematopoietic stem cells towards precocious myeloid differentiation at the expense of self-renewal. Nat. Cell Biol..

[132] Pronk CJ, Veiby OP, Bryder D, Jacobsen SEW (2011). Tumor necrosis factor restricts hematopoietic stem cell activity in mice: involvement of two distinct receptors. J. Exp. Med..

[133] Makris S (2022). Immune function and dysfunction are determined by lymphoid tissue efficacy. Dis. Model. Mech..

[134] Jung EM (2020). Association between prenatal exposure to PM2. 5 and the increased risk of specified infant mortality in South Korea. Environ. Int.

[135] Ortigoza, A. et al. Association between ambient PM2· 5 and under-5, infant, and child mortality in Latin America, 2010–15: a longitudinal analysis. **5**, S16 (2021).

[136] Bo Y (2021). Associations of reduced ambient PM2. 5 level with lower plasma glucose concentration and decreased risk of type 2 diabetes in adults: a longitudinal cohort study. Am. J. Epidemiol..

[137] Hayes RB (2020). PM2. 5 air pollution and cause-specific cardiovascular disease mortality. Int J. Epidemiol..

[138] Bowe B (2018). Particulate matter air pollution and the risk of incident CKD and progression to ESRD. J. Am. Soc. Nephrol..

[139] Lee J-TJC, Pediatrics E (2021). Review of epidemiological studies on air pollution and health effects in children. Clin. Exp. Pediatr..

[140] Li G, Li L, Liu D, Qin J, Zhu HJSR (2021). Effect of PM2. 5 pollution on perinatal mortality in China. Sci. Rep..

[141] Alman BL (2019). Associations between PM2. 5 and risk of preterm birth among liveborn infants. Ann. Epidemiol..

[142] Tao R-j (2020). PM2. 5 compromises antiviral immunity in influenza infection by inhibiting activation of NLRP3 inflammasome and expression of interferon-β. Mol. Immunol..

[143] Prada D, López G, Solleiro-Villavicencio H, Garcia-Cuellar C, Baccarelli AA (2020). Molecular and cellular mechanisms linking air pollution and bone damage. Environ. Res.

[144] Qiu Y-N (2019). PM2. 5 induces liver fibrosis via triggering ROS-mediated mitophagy. Ecotoxicol. Environ. Saf..

[145] Li R, Zhou R, Zhang J (2018). Function of PM2. 5 in the pathogenesis of lung cancer and chronic airway inflammatory diseases. Oncol. Lett..

[146] Chen S (2020). Effect of PM2. 5 on macrosomia in China: A nationwide prospective cohort study. Pediatr. Obes..

[147] Nääv Å (2020). Urban PM2. 5 induces cellular toxicity, hormone dysregulation, oxidative damage, inflammation, and mitochondrial interference in the HRT8 trophoblast cell line. Front Endocrinol. (Lausanne).

[148] Zhou S (2020). Ovarian dysfunction induced by chronic whole‐body PM2. 5 exposure. Small.

[149] int/mediacentre/factsheets/fs313/en/, W.H.O.J.R.f.W.H.O.A.f.h.w.w. Ambient (outdoor) air quality and health. 2018 (2016).

[150] Wu J (2021). The association between long-term exposure to ambient air pollution and bone strength in China. J. Clin. Endocrinol. Metab..

[151] Yang Y (2023). Ambient air pollution, bone mineral density and osteoporosis: results from a national population-based cohort study. Chemosphere.

[152] Qiao D (2020). Long-term exposure to air pollution might increase prevalence of osteoporosis in Chinese rural population. Environ. Res.

[153] Yu P (2022). Associations between long-term exposure to PM2. 5 and site-specific cancer mortality: A nationwide study in Brazil between 2010 and 2018. Environ. Pollut..

[154] Morales-Ancajima VC (2019). Increased outdoor PM2. 5 concentration is associated with moderate/severe anemia in children aged 6–59 months in Lima, Peru. J. Environ. Public Health.

[155] Honda T, Pun VC, Manjourides J, Suh H (2017). Anemia prevalence and hemoglobin levels are associated with long-term exposure to air pollution in an older population. Environ. Int..

[156] Visani G (2016). Environmental nanoparticles are significantly over-expressed in acute myeloid leukemia. Leuk. Res..

[157] Giannoni P (2021). Chronic lymphocytic leukemia cells impair osteoblastogenesis and promote osteoclastogenesis: role of TNFα, IL-6 and IL-11 cytokines. Haematologica.

[158] Wells A (2017). Systemic IL-6 effector response in mediating systemic bone loss following inhalation of organic dust. J. Interferon Cytokine Res..

[159] Calderón-Garcidueñas L (2013). Exposure to urban air pollution and bone health in clinically healthy six-year-old children. Arh. Hig. Rada Toksikol..

[160] Chen Y (2018). Probucol protects circulating endothelial progenitor cells from ambient PM2. 5 damage via inhibition of reactive oxygen species and inflammatory cytokine production in vivo. Exp. Ther. Med..

[161] Liberda EN (2010). Exposure to inhaled nickel nanoparticles causes a reduction in number and function of bone marrow endothelial progenitor cells. Inhal. Toxicol..

[162] Haberzettl P (2012). Exposure to ambient air fine particulate matter prevents VEGF-induced mobilization of endothelial progenitor cells from the bone marrow. Environ. Health Perspect..

[163] O’Toole TE (2010). Episodic exposure to fine particulate air pollution decreases circulating levels of endothelial progenitor cells. Circ. Res.

[164] Yamaguchi M, Kashiwakura I (2013). Role of reactive oxygen species in the radiation response of human hematopoietic stem/progenitor cells. PLoS One.

[165] Rodrigues-Moreira S (2017). Low-dose irradiation promotes persistent oxidative stress and decreases self-renewal in hematopoietic stem cells. Cell Rep..

[166] Eliasson P (2010). Hypoxia mediates low cell-cycle activity and increases the proportion of long-term–reconstituting hematopoietic stem cells during in vitro culture. Exp. Hematol..

[167] Ito K (2004). Regulation of oxidative stress by ATM is required for self-renewal of haematopoietic stem cells. Nature.

[168] Renzi M (2020). Short-term exposure to PM2. 5 and risk of venous thromboembolism: A case-crossover study. Thromb. Res.

[169] Cliff R (2016). Effect of diesel exhaust inhalation on blood markers of inflammation and neurotoxicity: a controlled, blinded crossover study. Inhal. Toxicol..

[170] Orona NS (2016). Acute exposure to Buenos Aires air particles (UAP-BA) induces local and systemic inflammatory response in middle-aged mice: A time course study. Environ. Pollut..

[171] Park S-R (2021). The impact of fine particulate matter (PM) on various beneficial functions of human endometrial stem cells through its key regulator SERPINB2. Exp. Mol. Med.

